# Supplementary material for: Correlation between varus-type knee osteoarthritis severity and hindfoot alignment: Analysis of radiographs in the long-leg weight-bearing anteroposterior view
Source: PLoS One. 2025 Jun 3;20(6):e0324974. doi: 10.1371/journal.pone.0324974 (PMC12133005; doi:10.1371/journal.pone.0324974)
Supplement: Attachment — Submitted filename: Answer for Journal Requirements 17-August-2024 Ver.2.docx [file pone.0324974.s002.docx]

Response to reviewer comments

Dear Reviewers,

We would like to thank the reviewers for your time and efforts in reviewing our manuscript and for providing comments, which have considerably helped us improve our manuscript. We have made revisions based on your comments and have provided our point-by-point responses below. We hope that our responses and revisions appropriately address your comments.

1. Is the manuscript technically sound, and do the data support the conclusions?

Response: In this study, radiographs were taken at the same facility using identical equipment and consistent conditions (see page 5). The sample sizes were determined based on a previous study, with references cited as numbers 3, 4, 5, and 6 (see page 15). Replication was assessed using intraclass correlation coefficients (ICC).

2. Has the statistical analysis been performed appropriately and rigorously?

Response: In this study, we utilized one-way analysis of variance (ANOVA) (see pages 8 and 9).

3. Have the authors made all data underlying the findings in their manuscript fully available?

Response: Yes, we have fully disclosed all the data and are attaching the data used in this study.

4. Is the manuscript presented in an intelligible fashion and written in standard English?

Response: The manuscript has been re-checked for proper language and grammar and to ensure that the text is written in standard English.

5. Review Comments to the Author

Please use the space provided to explain your answers to the questions above. You may also include additional comments for the author, including concerns about dual publication, research ethics, or publication ethics. (Please upload your review as an attachment if it exceeds 20,000 characters)

Reviewer #1: Thank you very much for allowing me to review your manuscript. I appreciate the effort you have made performing this study and submitting it to PLOS ONE. You mentioned that the present study showed a positive correlation up to grade II of K-L classification of varus-type knee OA and no positive correlation at grade III.

What about the correlation at grade 4?

In addition, with your results and conclusion, do you think you can add anything new to the science?

Response: In Kellgren–Lawrence (K–L) classification grade IV, there was an increase in the TCA angle; however, this cannot be considered a positive correlation. Based on the tibial calcaneal angle (TCA) results, we believe that the valgus alignment of the subtalar joint may serve as a compensatory mechanism in K–L classification grade II as knee osteoarthritis (KOA) varus progresses. However, in cases with K–L classification grade III or higher, the subtalar joint's compensatory function may not be sufficient to keep pace with the disease progression.

Reviewer #2: This manuscript contains several issues that require correction and clarification:

Abstract:

Rewrite the abstract of the article in a structured format according to the journal's guidelines.

Response: Based on your suggestion, the abstract has been revised in a structured format now.

Methodology:

Provide a detailed explanation of the study design in the methodology section.

Clearly state whether the study is retrospective or prospective, case-control or cohort, etc.

Response: The study design of this research is a retrospective case-control study. This has been specified in the Methods section now.

Explicitly define the inclusion and exclusion criteria for the study.

Justify why LL-WB AP view technique X-rays were taken in patients with soft tissue inflammation.

Response: The inclusion and exclusion criteria for the study are detailed in the first paragraph of the section titled “Study population and where you specify inclusion and exclusion criteria” (page 5).

The long-leg weight-bearing anteroposterior (LL-WB AP) view was utilized in patients with soft tissue conditions, such as anterior cruciate ligament injuries, to assess the severity of knee deformity in K–L Grade 0 patients. This approach was consistent with the imaging method used for other K–L grades.

Variables:

Cite the studies that introduced the angles variables measured in this study as references.

Response: The references for the femorotibial angle (FTA) are listed as numbers 1, 23, and 24 (pages 15 and 19). The references for TAS are numbers 25 and 26 (page 19). The reference for talar dome during weight-bearing (TTW) is number 27 (page 19). To the best of our knowledge, the definitions and angle measurements for the TCA are unique to this study.

6. PLOS authors have the option to publish the peer review history of their article (what does this mean?). If published, this will include your full peer review and any attached files.

If you choose “no”, your identity will remain anonymous but your review may still be made public.

Do you want your identity to be public for this peer review? For information about this choice, including consent withdrawal, please see our Privacy Policy.

Response: Yes.
