# Supplementary material for: Correlation between varus-type knee osteoarthritis severity and hindfoot alignment: Analysis of radiographs in the long-leg weight-bearing anteroposterior view
Source: PLoS One. 2025 Jun 3;20(6):e0324974. doi: 10.1371/journal.pone.0324974 (PMC12133005; doi:10.1371/journal.pone.0324974)
Supplement: Attachment — Submitted filename: Response to Reviewers 05-April-2025.docx [file pone.0324974.s003.docx]

Response to reviewer comments

Dear Reviewers,

We would like to thank the reviewers for your time and efforts in reviewing our manuscript and for providing comments, which have considerably helped us improve our manuscript. We have made revisions based on your comments and have provided our point-by-point responses below. We hope that our responses and revisions appropriately address your comments.

3. Has the statistical analysis been performed appropriately and rigorously?

Reviewer #3: I Don't Know.

Author #3: Thank you very much for allowing me to review your manuscript. I used ANOVA to assess differences between the stage and control groups in the T–T classification at the TCA, and Post-hoc analysis was performed using the Bonferroni test for multiple comparisons (please to see page 8). Based on the results of the analysis of variance, the probability of a significant difference between grade 2 and grade 3 of the K-L classification was determined to be 0.05 or less, and a significant difference was recognised. The results of the analysis of variance for TCA between each K-L classification are attached.

The variables are as follows: variable = TCA, independent variables = age/height/weight/BMI/K-L classification

| K-Lgrade 0+1/2 |  |  |  |  |  |
| --- | --- | --- | --- | --- | --- |
|  | sum of spuares | degree of freedom | mean spuares | F-measure | significance level |
| regression | 109.798 | 6 | 18.3 | 1.086 | 0.381 |
| residual | 994.156 | 59 | 16.85 |  |  |
| total | 1103.955 | 65 |  |  |  |
|  |  |  |  |  |  |
| K-Lgrade 2/3 |  |  |  |  |  |
|  | sum of spuares | degree of freedom | mean spuares | F-measure | significance level |
| regression | 228.129 | 6 | 38.022 | 2.422 | 0.033 |
| residual | 1302.86 | 83 | 15.697 |  |  |
| total | 1530.989 | 89 |  |  |  |
|  |  |  |  |  |  |
| K-Lgrade 3/4 |  |  |  |  |  |
|  | sum of spuares | degree of freedom | mean spuares | F-measure | significance level |
| regression | 69.041 | 6 | 11.507 | 0.732 | 0.625 |
| residual | 1352.078 | 86 | 15.722 |  |  |
| total | 1421.118 | 92 |  |  |  |

6. Review Comments to the Author

Reviewer #6: Please use the space provided to explain your answers to the questions above. You may also include additional comments for the author, including concerns about dual publication, research ethics, or publication ethics. (Please upload your review as an attachment if it exceeds 20,000 characters)

Author #6: First of all, thank you for pointing out the corrections. I have made the corrections by adding different colors.

Next, I will answer your question. My answer is “By studying the effects of knee osteoarthritis on the talocrural joint and sub-talar joint, I believe that it has become even clearer than in previous reports that it is possible to distinguish between the limit of conservative treatment for osteoarthritis and the timing of surgical treatment intervention in order to preserve the hindfoot. This indicates the appropriate timing for both conservative and surgical treatment, and I believe that it shows the possibility of providing more appropriate treatment by reducing the disadvantages and unnecessary physical and financial burdens on patients.”.

Do you want your identity to be public for this peer review? For information about this choice, including consent withdrawal, please see our Privacy Policy.

Author response: Yes.

Best regards

Yusuke Ozaki

Orthopaedic Department, Nara Medical.University, Kashihara city, Nara prefecture, Japan
